# Polariton hybridization phenomena on near-field radiative heat transfer in periodic graphene/*α*-MoO_3_ cells

**DOI:** 10.1515/nanoph-2022-0730

**Published:** 2023-04-10

**Authors:** Jihong Zhang, Bing Yang, Kezhang Shi, Haotuo Liu, Xiaohu Wu

**Affiliations:** School of Electromechanical and Automotive Engineering, Yantai University, Yantai 264005, Shandong, P. R. China; Centre for Advanced Laser Manufacturing (CALM), School of Mechanical Engineering, Shandong University of Technology, Zibo 255000, P. R. China; Centre for Optical and Electromagnetic Research, National Engineering Research Center for Optical Instruments, Zhejiang University, Hangzhou 310058, P. R. China; Shandong Institute of Advanced Technology, Jinan 250100, Shandong, P. R. China; School of Energy Science and Engineering, Harbin Institute of Technology, Harbin 150001, P. R. China

**Keywords:** *α*-MoO_3_, graphene, hyperbolic phonon polaritons, near-field radiative heat transfer, polariton hybridization phenomena, surface plasmon polaritons

## Abstract

Coupling of surface plasmon polaritons (SPPs) supported by graphene and hyperbolic phonon polaritons (HPPs) supported by hyperbolic materials (HMs) could effectively promote photon tunneling, and hence the radiative heat transfer. In this work, we investigate the polariton hybridization phenomena on near-field radiative heat transfer (NFRHT) in multilayer heterostructures, which consist of periodic graphene/*α*-MoO_3_ cells. Numerical results show that increasing the graphene/α-MoO_3_ cells can effectively enhance the NFRHT when the vacuum gap is less than 50 nm, but suppresses the enhanced performance with larger gap distance. This depends on the coupling of SPPs and HPPs in the periodic structure, which is analyzed by the energy transmission coefficients distributed in the wavevector space. The influence of the thickness of the α-MoO_3_ film and the chemical potential of graphene on the NFRHT is investigated. The findings in this work may guide designing high-performance near-field energy transfer and conversion devices based on coupling polaritons.

## 1 Introduction

Near-field radiative heat transfer (NFRHT) has been demonstrated to exceed the blackbody limit by several orders of magnitude [1–13]. When the gap distance of two objects is less than characteristic thermal wavelength, coupling effect of the evanescent waves will provide an efficient channel for photon transport, making the near-field radiative heat flux (NFRHF) greatly enhanced [14–28]. Such huge radiative heat flux has promising applications in noncontact refrigeration [29], thermal rectifications [30, 31], and thermophotovoltaics [32–36].

Previous studies of the NFRHT based on hyperbolic materials (HMs) show that the NFRHF can be promoted in a wide frequency range owing to the excitation of hyperbolic phonon polaritons (HPPs) [37–43]. HMs are a kind of materials with opposite signs of the permittivity components and exhibit superior hyperbolic dispersion properties [44–49]. Specifically, the natural HMs have remarkable advantages because of manifest hyperbolic dispersion without designing into nanostructures. *α*-MoO_3_ is a natural biaxial hyperbolic crystal with three wide bands [50]. Liu et al. have proposed a near-field radiative modulator, which demonstrated that effective modulation of NFRHT can be achieved by mechanical rotation of a hyperbolic material [51]. Wu et al. have exhibited that the NFRHT between two bulk α-MoO_3_ is much larger than that between two hBN [52]. However, the above studies mainly focus on the NFRHT between single materials and are less involved in composite structures based on HMs.

Graphene is an excellent two-dimensional material that can provide lots of resonances to facilitate photon tunneling [53]. It has been demonstrated that surface plasmon polaritons (SPPs) supported by graphene could couple HPPs in HMs to impact NFRHT [54–57]. In fact, the NFRHT between single-layer HMs covered by graphene has been thoroughly studied [58, 59]. Particularly, it is demonstrated that the single-cell structure is limited in both enhancement and modulation of NFRHT. Recent studies on the multilayer graphene/hBN heterostructures show that the coupling of SPPs in graphene and HPPs in hBN has the potential to further enhance NFRHT [60, 61]. Compared with hBN, α-MoO_3_ has broader hyperbolic bandwidth and longer polariton lifetime [62, 63]. Such a wide hyperbolic band allows HPPs to be excited in a wider frequency range, greatly promoting the NFRHT. Moreover, owing to the strong directional of the HPPs excited in *α*-MoO_3_, the NFRHT exhibits highly anisotropic characteristics, which makes it possible to achieve the modulation of the NFRHT. However, the effect of polariton hybridization phenomena on NFRHT in periodic graphene/*α*-MoO_3_ heterostructures has rarely been investigated, and the underlying physical mechanism needs further discussion.

Here, we take periodic graphene/*α*-MoO_3_ cells as a platform, and discuss the polariton hybridization phenomena on NFRHT. The characteristics of NFRHT were analyzed and compared between multilayer structures for different units. The coupling of the SPPs and HPPs explains the underlying physical mechanism. The energy transmission coefficients in the wavevector space are analyzed. Finally, the influence of the thickness of the α-MoO_3_ film and the chemical potential of graphene on NFRHT is investigated.

## 2 Theory and method

**Figure 1:**
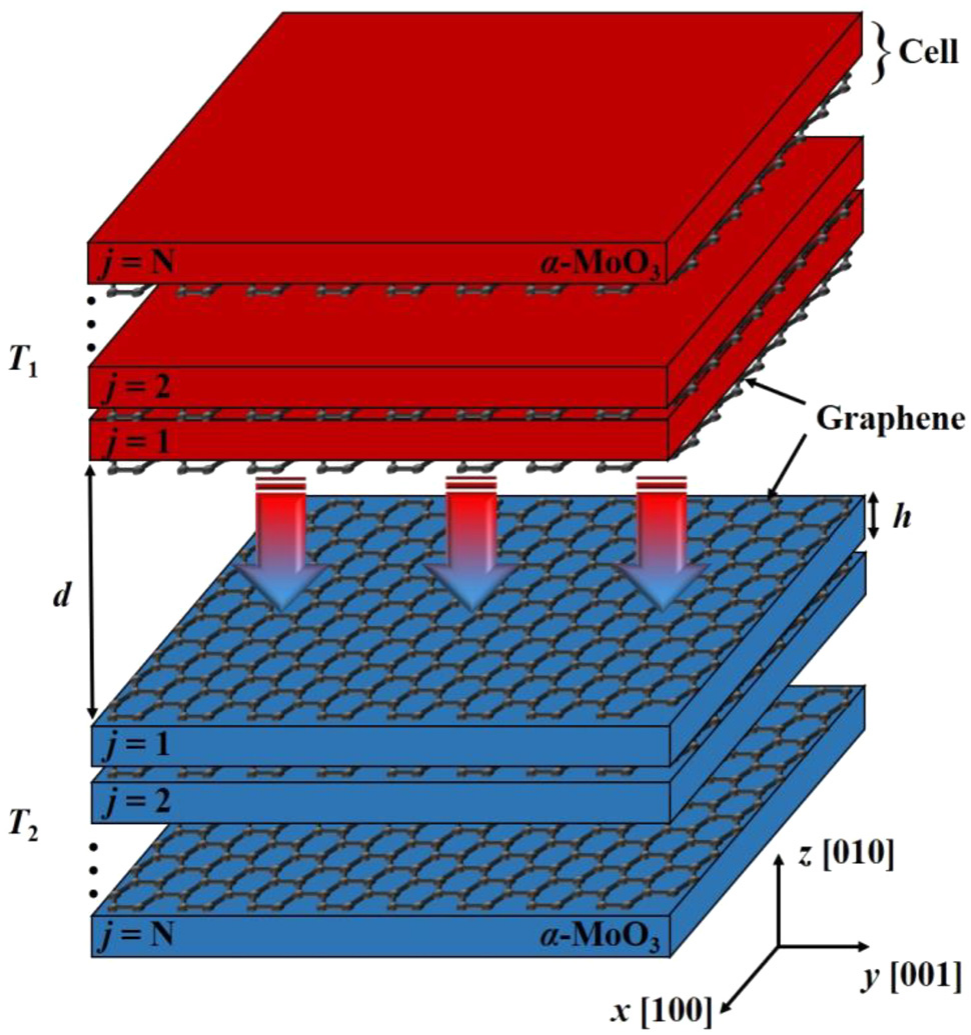
Schematic of the NFRHT between two periodic multilayer heterostructures. The number of cells is *N* and the cell configuration consists of *α*-MoO_3_ film covered by graphene.

Figure 1 shows the schematic of NFRHT between two periodic multilayer structures that consists of *α*-MoO_3_ film covered by graphene. The gap distance between the two objects is *d*, and *h* is the thickness of each *α*-MoO_3_ film. The temperature of emitter is *T*_1_ = 300 K, and temperature of receiver is *T*_2_ = 0 K. The number of cells is *N*. This work considers four different structures: single-cell, double-cell, five-cell, and ten-cell. The NFRHT in this work is along the [010] crystal direction of *α*-MoO_3_.

The permittivity of *α*-MoO_3_ is given by:

(1) (1) $$\varepsilon_{m}=\varepsilon_{\infty ,m}\left(1+\frac{\omega_{\text{LO},m}^{2}-\omega_{\text{TO},m}^{2}}{\omega_{\text{TO},m}^{2}-\omega^{2}+j\Gamma_{m}\omega }\right),\;m=x,y,z.$$

**Figure 2:**
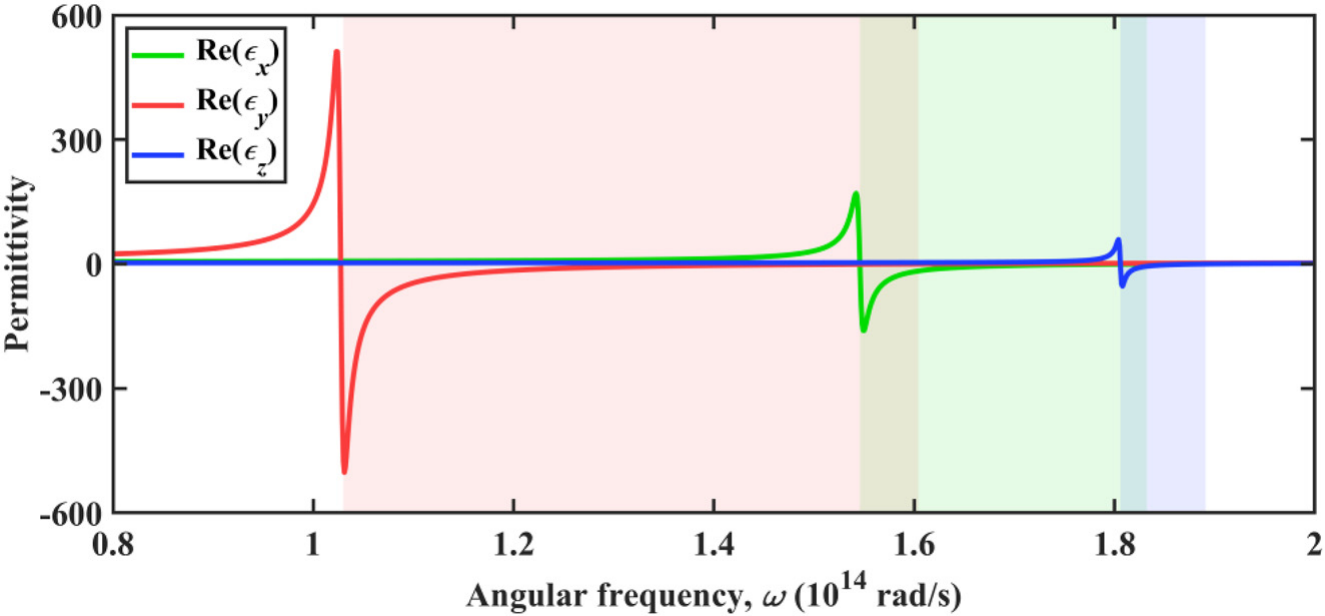
The real permittivity components of *α*-MoO_3_ vary in angular frequency. Hyperbolic bands are shaded in different colors.

Detailed parameters can be found in Ref. [64]. Figure 2 shows real permittivity components of *α*-MoO_3,_ and the shaded area represents three different hyperbolic bands. Graphene is an excellent two-dimensional material [65, 66]. In this work, to simplify the computational model, we ignore the effect of the background [67]. Thus, the conductivity of graphene is modelled by [58]:

(2) $$\sigma_{s}=\frac{{\text{e}}^{2}\mu }{\pi \hbar^{2}}\frac{\tau }{1-j\omega \tau }.$$

Detailed parameters can be found in Ref. [68]. In the calculation, the graphene is modeled as a layer of thickness Δ = 0.3 nm with an effective permittivity $\varepsilon_{\text{eff,G}}=1+j\sigma_{s}/\left(\varepsilon_{0}\omega \Delta \right)$ [69].

The NFRHF can be described as [70]:

(3) $$Q=\frac{1}{8\pi^{3}}\overset{\infty }{\underset{0}{\int }}\left[\Theta \left(\omega ,T_{1}\right)-\Theta \left(\omega ,T_{2}\right)\right]\text{d}\omega \overset{2\pi }{\underset{0}{\int }}\overset{\infty }{\underset{0}{\int }}\xi \left(\omega ,\beta ,\phi \right)\beta d\beta d\phi ,$$

where $\xi \left(\omega ,\beta ,\phi \right)$ is known as the energy transmission coefficient, which is given by [70]:

(4) $$\xi \left(\omega ,\beta ,\phi \right)=\left\{\begin{array}{c} \mathrm{Tr}\left[\left(I-R_{2}^{*}R_{2}-T_{2}^{*}T_{2}\right)D\left(I-R_{1}^{*}R_{1}-T_{1}^{*}T_{1}\right)D^{*}\right],\;\beta <k_{0}\;\\ \mathrm{Tr}\left[\left(R_{2}^{*}-R_{2}\right)D\left(R_{1}-R_{1}^{*}\right)D^{*}\right]{\text{e}}^{-2\left|k_{z}\right|d},\;\beta >k_{0}\; \end{array}\right.,$$

where **R** and **T** represent Fresnel’s reflection/transmission coefficients for *p* or *s* polarization, which can be expressed as:

(5) $$R_{1\text{,}2}=\left[\begin{array}{cc} r_{ss}^{1,2} & r_{sp}^{1,2}\\ r_{ps}^{1,2} & r_{pp}^{1,2} \end{array}\right],\;T_{1\text{,}2}=\left[\begin{array}{cc} t_{ss}^{1,2} & t_{sp}^{1,2}\\ t_{ps}^{1,2} & t_{pp}^{1,2} \end{array}\right],$$

where **D** is a Fabry–Perot-like denominator matrix and is obtained by $D={\left(I-R_{1}R_{2}{\text{e}}^{2jk_{z}d}\right)}^{-1}$ . The detailed calculation process and method can be found in Ref. [52].

## 3 Results and discussion

**Figure 3:**
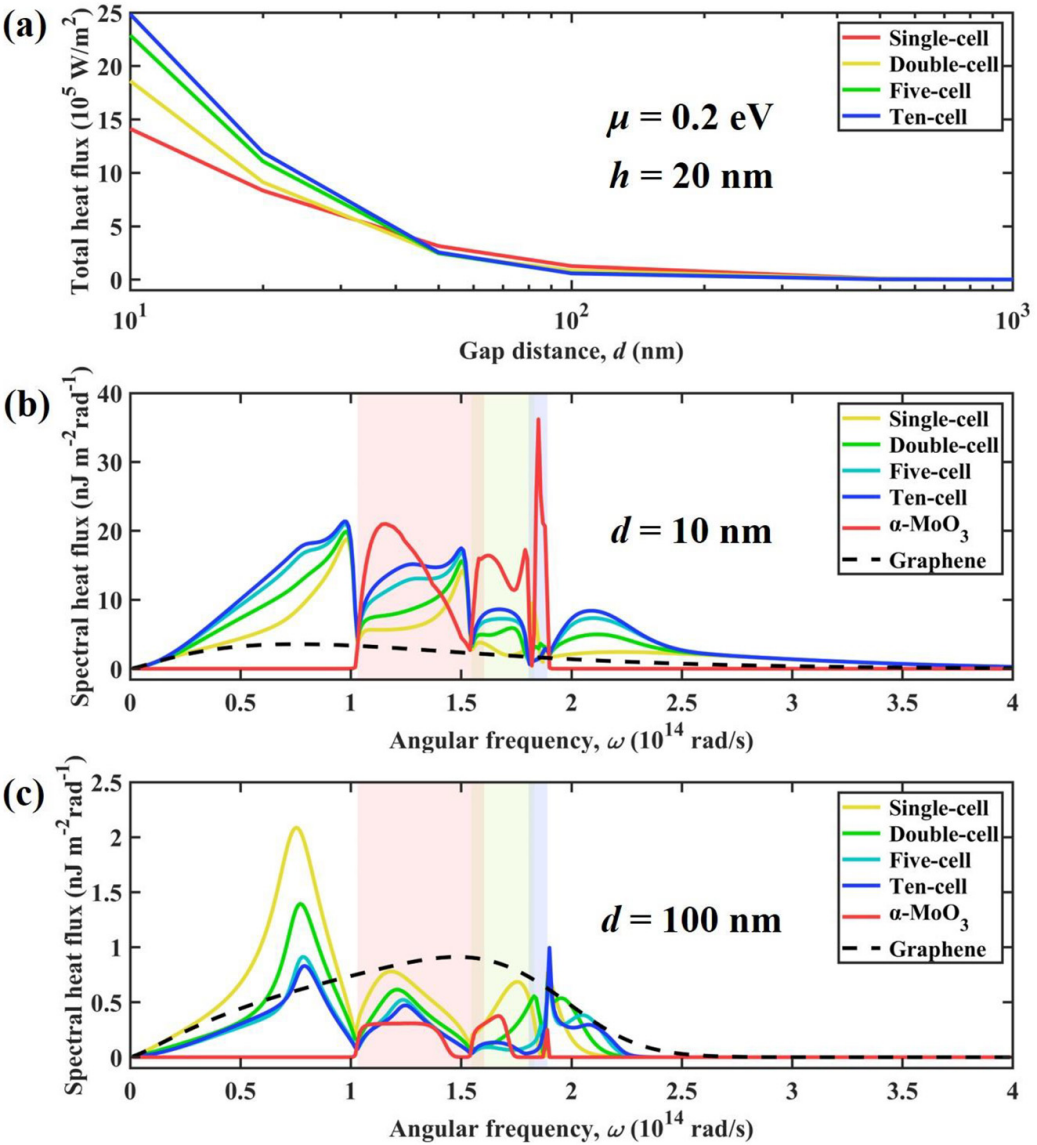
NFRHF varies in gap distance for different configurations. (a) Total heat flux varies in vacuum gap from 10 nm to 1000 nm for four configurations. (b) Spectral heat flux varies in angular frequency for six configurations at *d* = 10 nm. (c) Spectral heat flux varies in angular frequency for six configurations at *d* = 100 nm. The *α*-MoO_3_ in the legend denotes the *α*-MoO_3_-cell configuration. The graphene in the legend denotes the graphene-cell configuration.

To start, we calculate the NFRHT between periodic multilayer graphene/*α*-MoO_3_ heterostructures for different cells, which is illustrated in Figure 3(a). The heat flux drops sharply when *d* increases due to the evanescent effects. When *d* < 50 nm, *Q* increases with larger number of graphene/*α*-MoO_3_ cells in configurations. The ten-cell configuration shows the largest radiative heat flux compared to that of the other three configurations (single-cell, double-cell, and five-cell). Particularly, when the vacuum gap is 10 nm, the heat flux of ten-cell configuration reaches 2.49 × 10^6^ W/m^2^, which is 1.76-fold higher than that of the single-cell structure. This result can be confirmed by the variation of spectral heat flux with angular frequency in Figure 3(b). Note that the ten-cell configuration obviously exhibits the largest area between spectral heat flux and angular frequency. Moreover, to study the coupling effects of *α*-MoO_3_ and graphene, the spectral heat flux of graphene without *α*-MoO_3_ films (marked as graphene in the legend), and bare *α*-MoO_3_ films without graphene (marked as *α*-MoO_3_ in the legend) are shown in Figure 3(b). The spectral heat flux of the graphene-cell configuration covers a broadband, while the spectral heat flux of the *α*-MoO_3_-cell configuration is only covered from 1.03 × 10^14^ to 1.89 × 10^14^ rad/s. In addition, the spectral heat flux of the bare *α*-MoO_3_ film is much higher than that of the graphene sheet. The single-cell configuration represents the graphene-covered *α*-MoO_3_ film. The spectral heat flux of single-cell covers a broader frequency band over the Reststrahlen bands due to the broadband supported by graphene plasmons. The value of spectral heat flux of single-cell can beat that of the graphene sheet but is still smaller than that of the bare *α*-MoO_3_ within the Reststrahlen bands. The spectral heat flux in the Reststrahlen band of *α*-MoO_3_ films is suppressed by covering graphene. When more layers of graphene/*α*-MoO_3_ heterostructures are added to the configurations, the spectral heat flux will be enhanced by the strong hybrid/coupled multiple graphene SPPs and HPPs of *α*-MoO_3_ films, which directly explains the enhancement with the increase of the number of graphene-*α*-MoO_3_ cells in configurations.

However, when vacuum gap is larger than 50 nm, the heat flux of single-cell configuration shows the largest thermal radiation of NFRHT compared to the other three configurations. The variation of spectral heat flux with angular frequency can confirm the phenomenon in Figure 3(c), which shows the change phenomenon opposite to that at narrow gaps. Spectral heat flux gradually decreases with increase of graphene-*α*-MoO_3_ cells. This can be explained by the rapid attenuation of the coupling of SPPs and HPPs of the multilayer structure in the wavevector space due to the increased spacing [13, 60, 71, 72]. Since the thickness of each *α*-MoO_3_ layer is much smaller than the vacuum spacing, it makes the coupling of the total hybridization mode weaken or disappear. In addition, the optical/material loss of the multilayer structure has a greater negative influence on the polaritons mode than that of the single-layer structure, which ultimately makes the multilayer hybridization mode have no contribution in the larger transverse wavevector. Therefore, the NFRHT between single-layer structures is superior to that of multilayer structures.

**Figure 4:**
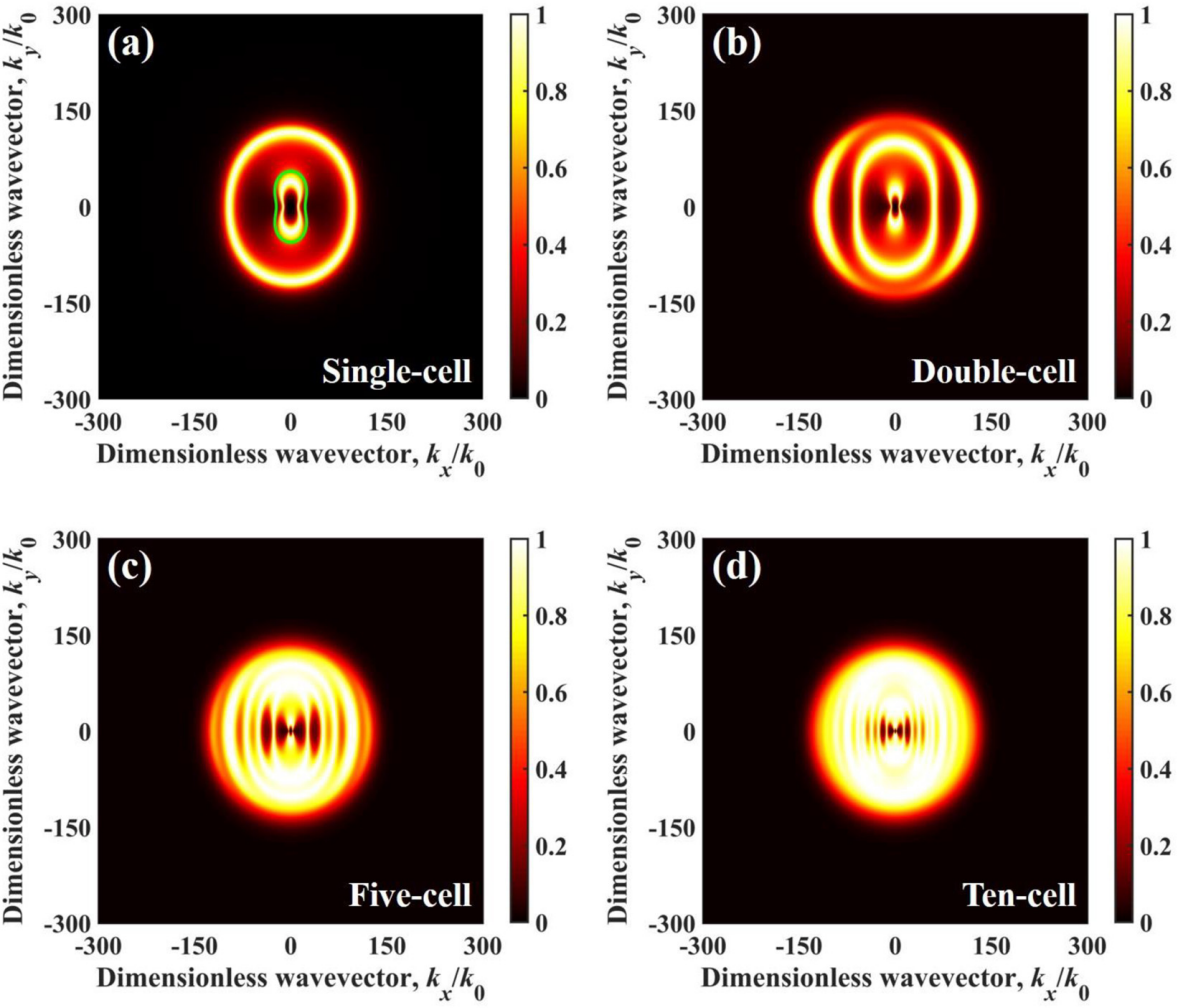
Energy transmission coefficient varies in dimensionless wavevector at 0.75 × 10^14^ rad/s and vacuum gap *d* = 10 nm for different configurations, (a) single-cell, (b) double-cell, (c) five-cell, and (d) ten-cell. The green solid line represents the dispersion relation.

Moreover, we calculated energy transmission coefficient distribution for four configurations of multilayer structure at angular frequency *ω* = 0.75 × 10^14^ rad/s, which is demonstrated in Figure 4. When the angular frequency is *ω* = 0.75 × 10^14^ rad/s, the permittivity of *α*-MoO_3_ is *ε*_*x*_ = 6.1193 + j0.0066, *ε*_*y*_ = 21.2119 + j0.1837, *ε*_*z*_ = 2.6852 + j0.0003. Because the conditions of *ε*_*x*_ > 0, *ε*_*y*_ > 0, *ε*_*z*_ > 0, the HPPs in *α*-MoO_3_ cannot be excited at this angular frequency. In particular, when the angular frequency is outside the hyperbolic band of *α*-MoO_3_, the main contribution of NFRHT between multilayer structures is the SPPs. The dispersion relation of single-cell structure can be described by [73]:

(6) (6) $$q=\frac{\rho }{k_{0}t}\left[\tan^{-1}\left(\frac{\rho \left(\varepsilon_{1}+jq\sigma Z_{0}\right)}{\varepsilon_{z}}\right)+\tan^{-1}\left(\frac{\varepsilon_{3}\rho }{\varepsilon_{z}}\right)+l\pi \right],$$

where $Z_{0}=\sqrt{\mu_{0}/\varepsilon_{0}}$ is the impedance of free space; *σ* is the conductivity of graphene. It is clear that the green solid dispersion curve matches well with the energy transmission coefficient in wavevector space, as shown in Figure 4(a). We found that as the number of cells increases, the area and intensity of excitation of SPPs increases in the wavevector space, which is responsible for the dramatic changes in the spectral heat flux at that frequency in Figure 3(b).

**Figure 5:**
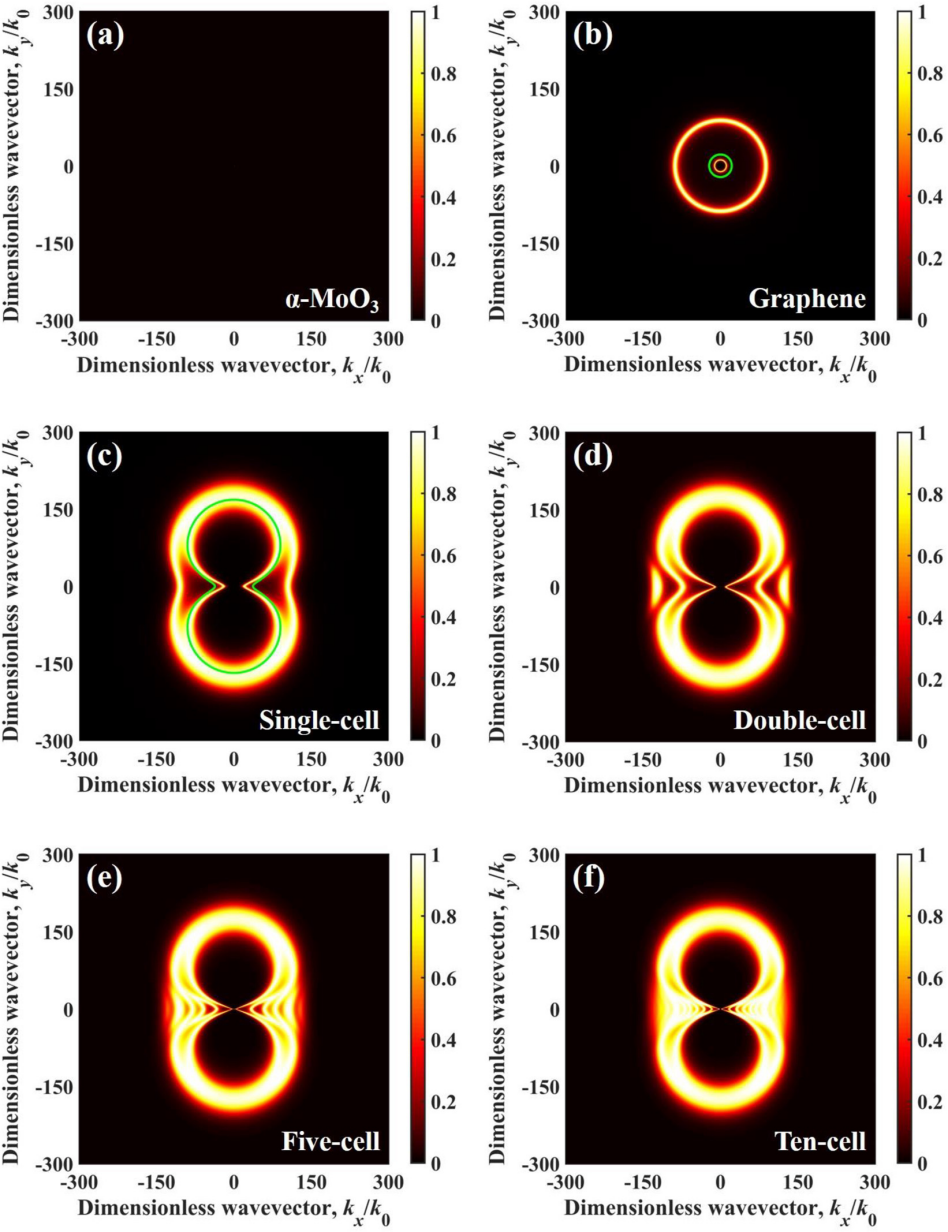
Energy transmission coefficient varies in dimensionless wavevector at 0.97 × 10^14^ rad/s and vacuum gap *d* = 10 nm for different configurations, (a) *α*-MoO_3_-cell configuration (*α*-MoO_3_ films without graphene), (b) graphene-cell configuration (single-cell without *α*-MoO_3_ films), (c) single-cell, (d) double-cell, (e) five-cell, and (f) ten-cell. The green solid line represents the dispersion relation.

There are several sharp peaks in the spectral heat flux curves at the 10 nm vacuum gap. The energy transmission coefficients are further analyzed at different angular frequencies for the underlying physics of the four configurations. At *ω* = 0.97 × 10^14^ rad/s outside the Reststrahlen band of *α*-MoO_3_, the permittivity of *α*-MoO_3_ is *ε*_*x*_ = 6.6731 + j0.0135, *ε*_*y*_ = 73.8813 + j4.3890, *ε*_*z*_ = 2.7317 + j0.0005, respectively. Since three real components of the permittivity of *α*-MoO_3_ are positive, there are no HPPs excited in *α*-MoO_3_ shown in Figure 5(a). For the suspended graphene sheets (Figure 5(b)), two concentric rings of coupled SPPs occur at *ω* = 0.97 × 10^14^ rad/s. The green solid line represents the dispersion relation, which can be described by [74]:

(7) $$q=\frac{\rho }{k_{0}t}\left[\arctan\left(\frac{\varepsilon_{1}\rho }{\varepsilon_{z}}\right)+\arctan\left(\frac{\varepsilon_{3}\rho }{\varepsilon_{z}}\right)+\pi l\right],\;l=0,1,2\cdots \;,$$

where $\rho =j\sqrt{\frac{\varepsilon_{z}}{\varepsilon_{x}\cos^{2}\varphi +\varepsilon_{y}\sin^{2}\varphi }}$ ; *φ* is the angle between the *x*-axis and in-plane component of the wavevector; Mediums 1 and 3 represent air (*ε*_1_ = *ε*_3_ = 1); *l* represents the excitation order; *t* is the thickness of the film. Because frequencies of the coupling polaritons are outside the hyperbolic band, the SPPs is the main reason of spectral heat flux variation. The SPPs in the structure (single-cell configuration) are excited like two connected rings shown in Figure 5(c), which is in an anisotropic-shape due the in-plane (*x*–*y* plane) anisotropic permittivity of the *α*-MoO_3_. In addition, the evolution process from Figure 5(c)–(f) demonstrates that the excitation of SPPs barely changes on upper and lower sides, but gradually increases on left and right sides. In other words, ten-cell configuration supports more continuous SPPs mode than other configurations on left and right sides. This explains why ten-cell configuration exhibits the highest peak than the other three configurations at *ω* = 0.97 × 10^14^ rad/s. A similar phenomenon of spectral heat flux in other regions of angular frequencies outside the Reststrahlen band of *α*-MoO_3_ can be explained similarly.

**Figure 6:**
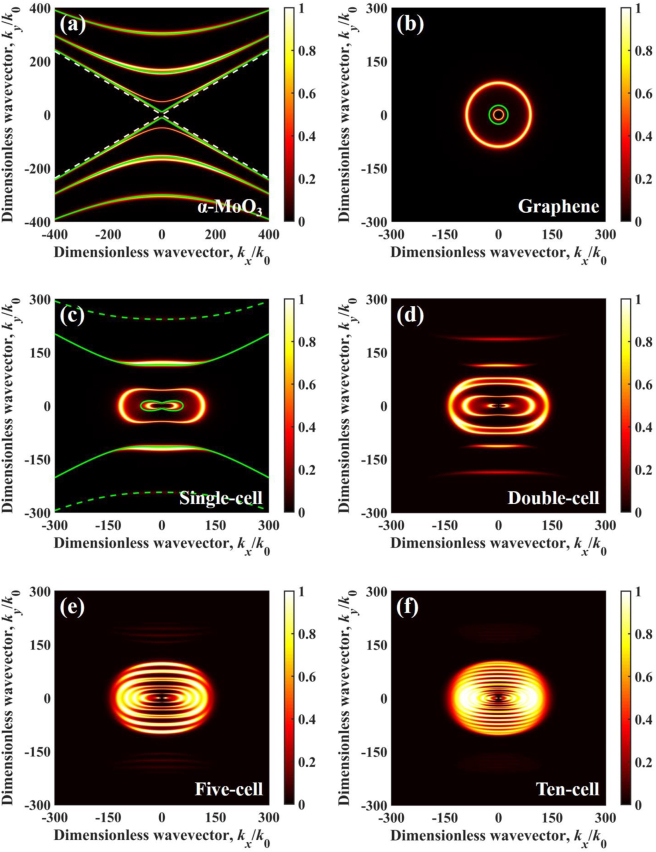
Energy transmission coefficient varies in dimensionless wavevector at 1.16 × 10^14^ rad/s and vacuum gap *d* = 10 nm for different configurations, (a) *α*-MoO_3_-cell configuration (*α*-MoO_3_ films without graphene), (b) graphene-cell configuration (single-cell without *α*-MoO_3_ films), (c) single-cell, (d) double-cell, (e) five-cell, and (f) ten-cell. The white dashed lines represent *k*_*y*_ = ±0.59*k*_*x*_. The green line represents the dispersion relation. Note that the purpose of using the dashed line in (c) is to avoid shading the energy transfer coefficient.

When *ω* = 1.16 × 10^14^ rad/s, permittivity of *α*-MoO_3_ is *ε*_*x*_ = 7.7096 + j0.0311, *ε*_*y*_ = −21.9670 + j0.8186, *ε*_*z*_ = 2.8018 + j0.0009, respectively. According to eight cases in Ref. [58], volume-confined hyperbolic polaritons (VHPs) are excited in *α*-MoO_3_ on the upper and lower sides (Figure 6(a)) onto the *k*_*x*_–*k*_*y*_ plane due to the signs of three permittivity components Re[*ε*_*x*_] > 0, Re[*ε*_*y*_] < 0, Re[*ε*_*z*_] > 0. The boundary lines can be represented by:

(8) $$k_{y}=\pm \sqrt{-\frac{\varepsilon_{x}}{\varepsilon_{y}}}k_{x}.$$

Therefore, the asymptotes are *k*_*y*_ = ±0.59*k*_*x*_, denoted as green dashed lines shown in Figure 6(a). Figure 6(b) shows SPPs excited in graphene-cell configuration at *ω* = 1.16 × 10^14^ rad/s. Particularly, discrete VHPs of a concave shape is supported by *α*-MoO_3_ (Figure 6(a)) and excited in a wide region of wavevector, while SPPs supported by graphene (Figure 6(b)) are excited in a small region of wavevector. It is clear that the dispersion curves match well with the energy transmission coefficients in wavevector space. In the case of single-cell configuration, HPPs couple with SPPs to form coupling polaritons with a weakened concave shape combined with a ring shape shown in Figure 6(c). These coupling polaritons are excited in a smaller region of wavevector than that of HPPs in Figure 6(a). As a result, spectral heat flux decreases at 1.16 × 10^14^ rad/s when *α*-MoO_3_ film is covered by graphene. In addition, more cell configurations could support more continuous coupling polaritons shown in Figure 6(d)–(f). Consequently, the ten-cell configuration exhibits the highest radiative heat flux than other three configurations at 1.16 × 10^14^ rad/s.

**Figure 7:**
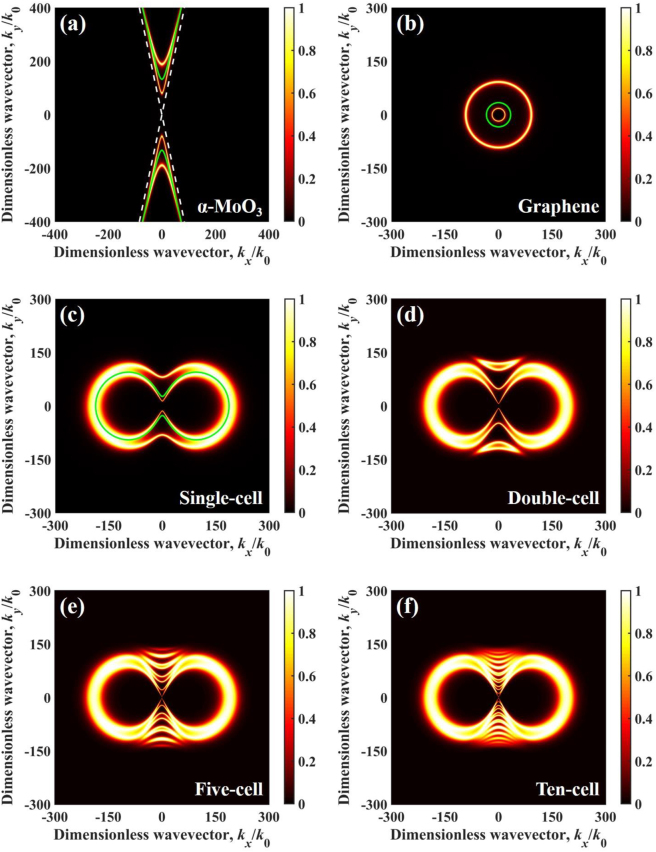
Energy transmission coefficient varies in dimensionless wavevector at 1.5 × 10^14^ rad/s and vacuum gap *d* = 10 nm for different configurations, (a) *α*-MoO_3_-cell configuration (*α*-MoO_3_ films without graphene), (b) graphene-cell configuration (single-cell without *α*-MoO_3_ films), (c) single-cell, (d) double-cell, (e) five-cell, and (f) ten-cell. The white dashed lines represent *k*_*y*_ = ±4.74*k*_*x*_. The green solid line represents the dispersion relation.

When *ω* = 1.5 × 10^14^ rad/s, the permittivity of *α*-MoO_3_ is *ε*_*x*_ = 31.6522 + j2.2487, *ε*_*y*_ = −1.4059 + j0.0625, *ε*_*z*_ = 3.1613 + j0.0043, respectively. VHPs are excited in *α*-MoO_3_ on upper and lower sides shown in Figure 7(a), and the asymptotes are *k*_*y*_ = ±4.74*k*_*x*_, denoted as green-dashed lines. VHPs supported by *α*-MoO_3_ shown in Figure 7(a) could couple with SPPs supported by graphene shown in Figure 7(b) to form the coupling polaritons shown in Figure 7(c). Compared to the VHPs in Figure 7(a), the coupling polaritons are in a smaller region on upper and lower sides and in wide region on left and right sides in wavevector space, which helps to enhance the spectral heat flux at 1.5 × 10^14^ rad/s. As the number of graphene-*α*-MoO_3_ cells increases, the coupling polaritons barely change on left and right sides, and gradually enhance on upper and lower sides. Consequently, ten-cell configuration exhibits the highest radiative heat flux than that of the other three configurations at 1.5 × 10^14^ rad/s. Furthermore, SPPs are highly enhanced in large areas, although VHPs are excited in limited azimuthal angles. Hence, the coupled polaritons make the spectral heat flux of the single-cell configuration much higher than that of the *α*-MoO_3_ cell configuration and the graphene cell configuration.

**Figure 8:**
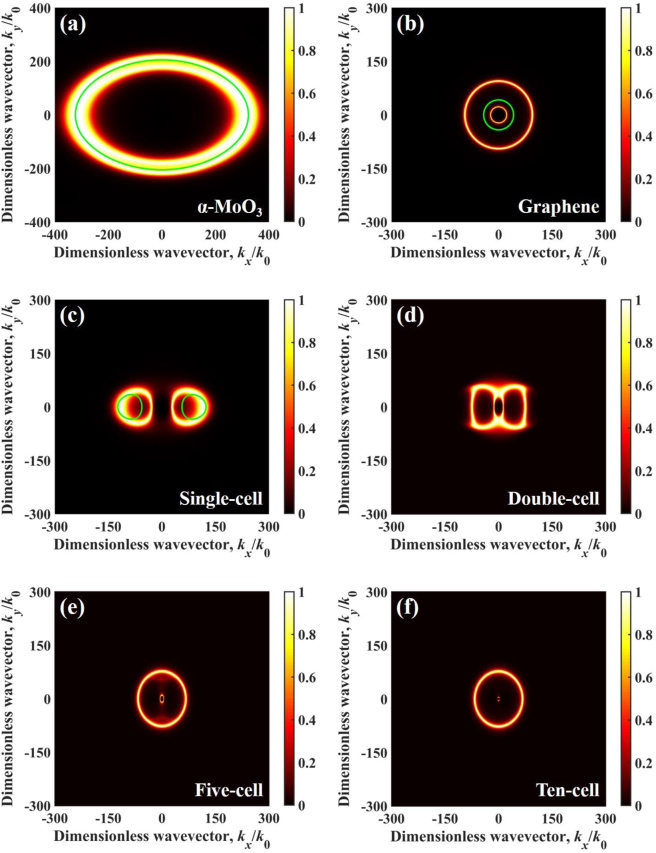
Energy transmission coefficient varies in dimensionless wavevector at 1.85 × 10^14^ rad/s and vacuum gap *d* = 10 nm for different configurations, (a) *α*-MoO_3_-cell configuration (*α*-MoO_3_ films without graphene), (b) graphene-cell configuration (single-cell without *α*-MoO_3_ films), (c) single-cell, (d) double-cell, (e) five-cell, and (f) ten-cell. The green solid line represents the dispersion relation.

At *ω* = 1.85 × 10^14^ rad/s located at the edge of the Reststrahlen band in *α*-MoO_3_, the permittivity of *α*-MoO_3_ is *ε*_*x*_ = 0.2547 + j0.0506, *ε*_*y*_ = 1.8660 + j0.0196, *ε*_*z*_ = −2.3527 + j0.2051, respectively. As demonstrated in Figure 8(a), VHPs are excited at all azimuthal angles within wide wavevectors, since the sign of *ε*_*z*_ is negative, and the signs of both *ε*_*x*_ and *ε*_*y*_ are positive. However, SPPs supported by graphene (Figure 8(b)) are excited in a small region of wavevectors. The dispersion curves in Figure 8(a) and (b) are calculated from Eq. (7). The coupling polaritons are excited in small wavevectors demonstrated in Figure 8(c). As the number of cell increases, the wavevector range of coupling polaritons excitation is further compressed. Consequently, the single-cell configuration shows the highest radiative heat flux than the other three configurations at 1.85 × 10^14^ rad/s.

**Figure 9:**
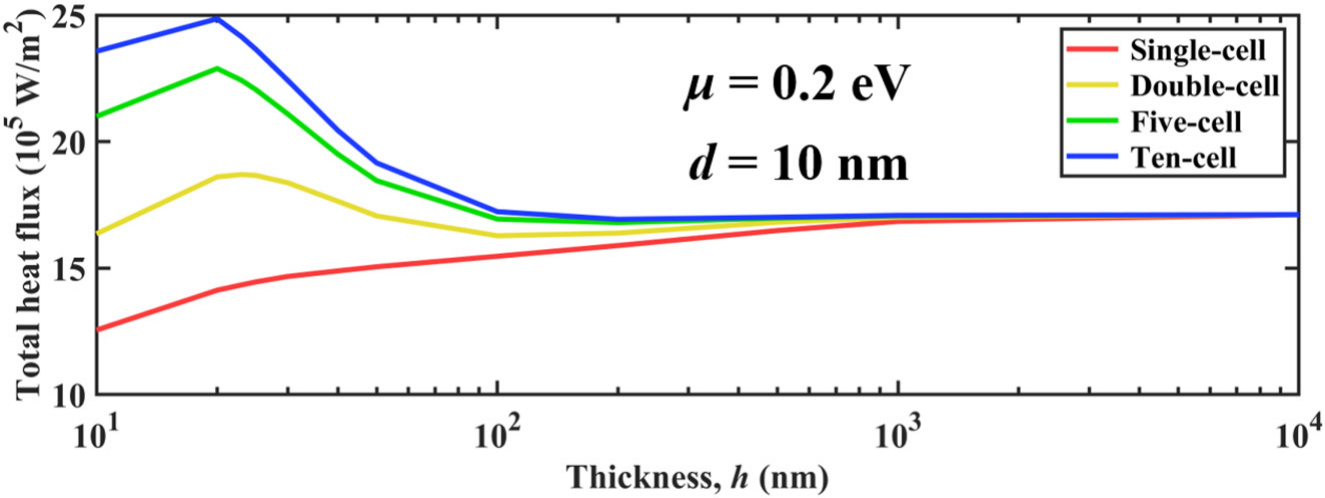
Total heat flux versus α-MoO_3_ thickness for four different configurations. The vacuum gap is 10 nm.

Moreover, we investigated the effect of the thickness of *α*-MoO_3_ on the NFRHT between multilayer graphene/*α*-MoO_3_ heterostructures, as shown in Figure 9. At the case of *μ* = 0.2 eV and *d* = 10 nm, the total heat flux increases significantly with the number of cells, especially when the *α*-MoO_3_ layer is very thin. When the thickness of *α*-MoO_3_ is 10 nm, the heat flux between ten-cell structures is 23.57 × 10^5^ W/m^2^, which is 87.81 % larger than that of single-cell structure (12.55 × 10^5^ W/m^2^). For the single-cell structure, the heat flux monotonically increases with the thickness of *α*-MoO_3_ film. However, the heat flux between the multi-cell structures increases and then decreases with the thickness of *α*-MoO_3_ film, which agrees well with that in multilayer graphene-hBN heterostructures in Ref. [60]. In addition, the effect of the number of cells on the heat flux decreases as the thickness of the *α*-MoO_3_ film increases. When the thickness of the *α*-MoO_3_ film is large enough (>1000 nm), the heat flux hardly varies with the number of cells.

**Figure 10:**
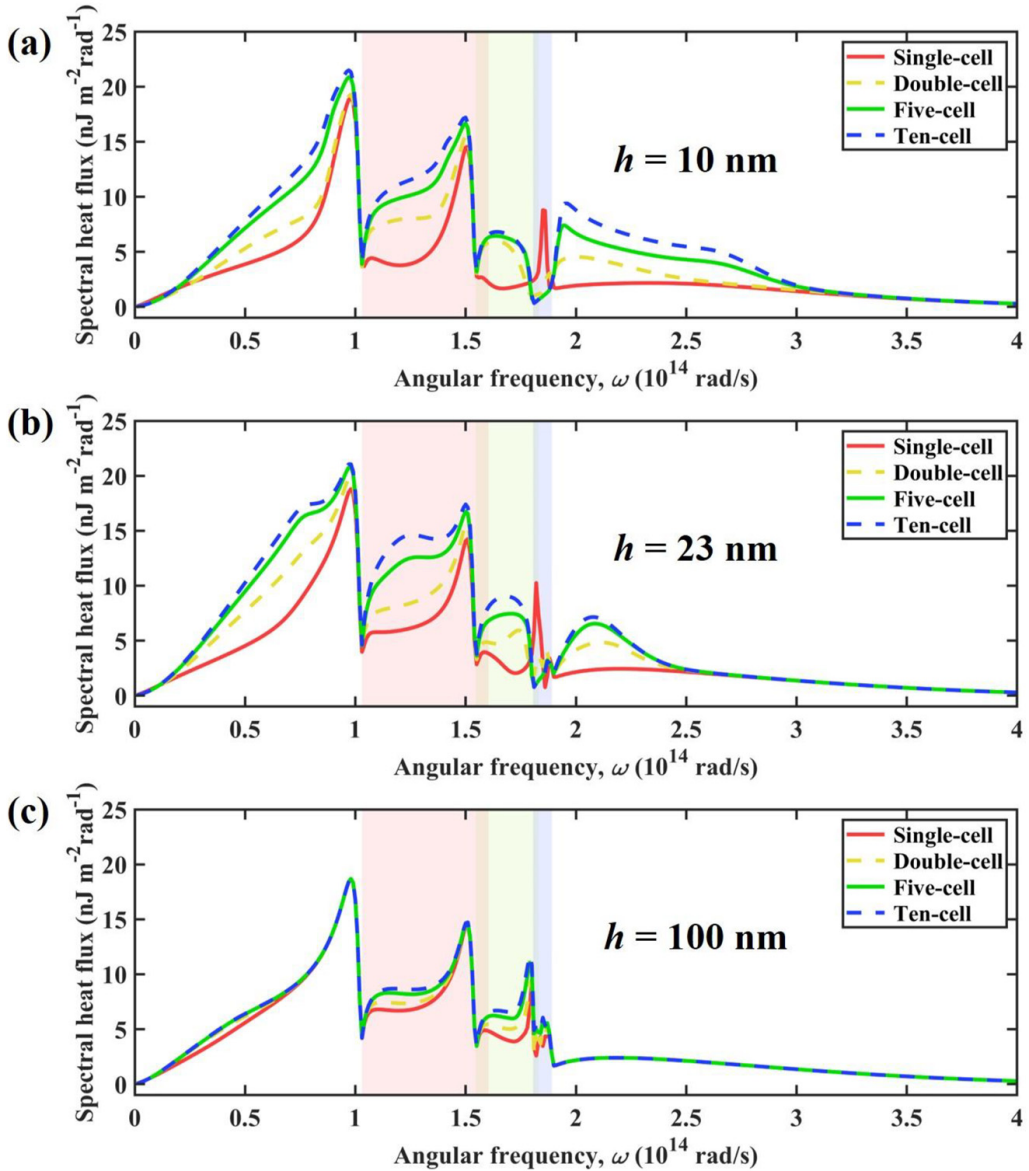
Spectral heat flux versus angular frequency for four different configurations at different thicknesses.

The spectral heat flux between multilayer graphene/*α*-MoO_3_ heterostructures in four configurations for different thicknesses of *α*-MoO_3_ film is calculated in Figure 10. In the case of *h* = 10 nm, the spectral heat flux changes drastically with the number of cells. When the frequency is outside the hyperbolic band of *α*-MoO_3_, the graphene-supported SPPs are the main reason for the variation of the spectral heat flux. Since the distance between graphene is small, increasing the number of units is beneficial to enhance the coupling of SPPs between different graphene, further promoting NFRHT. When the frequency is within the hyperbolic band of *α*-MoO_3_, the main reason for the spectral heat flux variation is the coupling of graphene-supported SPPs with *α*-MoO_3_-supported HPPs. When the number of cells increases, the coupled polaritons are further promoted, significantly enhancing the spectral heat flux. Notably, when the frequency is in the Band III hyperbolic region of *α*-MoO_3_, increasing the number of cells suppresses the NFRHT. The case of *h* = 23 nm is similar to that of *h* = 10 nm, differing only at high frequencies (2.5 × 10^14^ rad/s – 3 × 10^14^ rad/s), that is, the spectral heat flux is almost independent of the number of cells in that frequency range. When the thickness of *α*-MoO_3_ is 100 nm, the change in spectral heat flux occurs mainly within the hyperbolic band of *α*-MoO_3_. Increasing the number of cells promotes the coupling of SPPs and HPPs, and therefore the spectral heat flux is enhanced. The spectral heat flux varies little with the number of cells outside the hyperbolic band of *α*-MoO_3_, which is due to the large thickness of the *α*-MoO_3_ films, which weakens the coupling of SPPs between different graphene.

**Figure 11:**
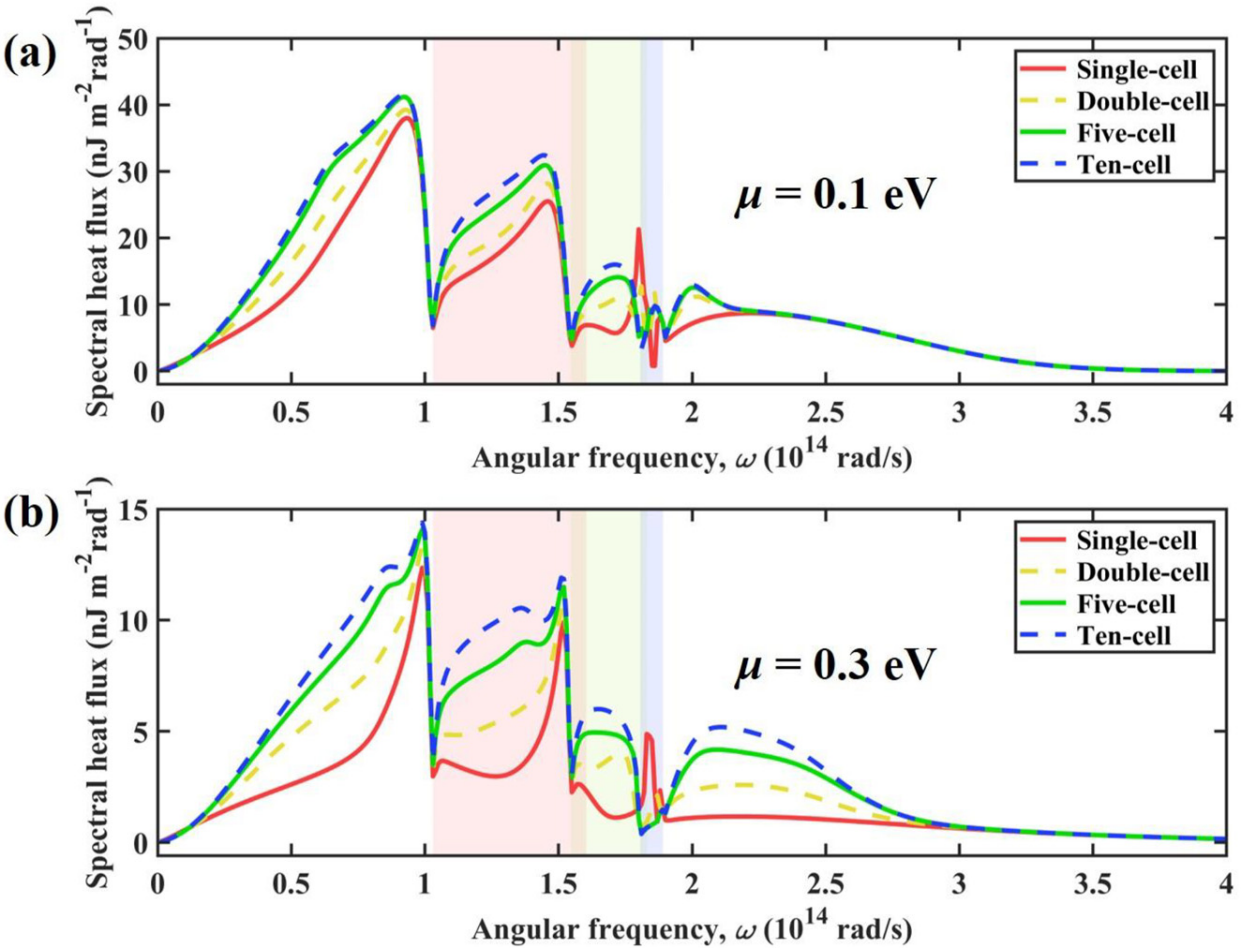
Spectral heat flux versus angular frequency for four configurations at different chemical potentials of graphene.

Finally, the effect of chemical potential of graphene on the NFRHT between multilayer graphene/*α*-MoO_3_ heterostructures is discussed, as shown in Figure 11. Note *d* = 10 nm and *h* = 20 nm. For the case of *μ* = 0.1 eV, Figure 11(a) shows that as the number of cells increases, the spectral heat flux increases significantly, especially in the hyperbolic band of *α*-MoO_3_ and in the low-frequency region (<1 × 10^14^ rad/s). For the ten-cell structure, the total heat flux is 47.65 × 10^5^ W/m^2^, which is 33.4 % more than that of the single-cell structure (35.72 × 10^5^ W/m^2^). When the chemical potential of graphene is *μ* = 0.3 eV, the NFRHT is significantly suppressed. We find the number of cells affects the spectral heat flux in the high-frequency region (>2 × 10^14^ rad/s). The total heat flux is 16.90 × 10^5^ W/m^2^ for the ten-cell structure, which is 115 % larger than that of the single-cell structure (7.86 × 10^5^ W/m^2^), which exhibits more dramatic variations of the spectral heat flux.

## 4 Conclusions

We theoretically investigate the effect of polariton hybridization phenomena on NFRHT, using periodic graphene/*α*-MoO_3_ cells as a platform. The radiative heat transfer characteristics of four different kinds of periodic cell structures are compared and analyzed. When the thickness of each *α*-MoO_3_ is fixed, our calculation shows that when the vacuum gap is less than 50 nm, adding graphene/α-MoO_3_ cells is effective in enhancing the NFRHT. However, when the vacuum gap is large, adding cells would suppress the NFRHT between multilayer structures with fixed thickness of each layer. The underlying physics can be explained by the coupling of SPPs and HPPs, which is equivalently illustrated by the energy transmission coefficients distribution in wavevector space. Moreover, we discuss the influence of the thickness of α-MoO_3_ on the NFRHT. The heat flux monotonously increases with increasing film thickness for single-cell structures, while it has an optimal value for other multi-cell structures. Finally, we investigate the effect of the chemical potential of graphene on the NFRHT. The findings of this work may help to design tunable near-field thermal radiation systems.
